# Validating dynamic time warping as a measure of gesture form similarity

**DOI:** 10.3758/s13428-026-02975-5

**Published:** 2026-04-10

**Authors:** Sho Akamine, Mark Dingemanse, Aslı Özyürek

**Affiliations:** 1 Max Planck Institute for Psycholinguistics, Wundtlaan 1, 6525 XD Nijmegen, The Netherlands; 2 Centre for Language Studies, Radboud University, Nijmegen, The Netherlands; 3 Donders Institute for Brain, Cognition and Behavior, Nijmegen, The Netherlands

**Keywords:** Dynamic time warping, Gesture form similarity, Multimodal language

## Abstract

Dynamic time warping (DTW) is a well-known algorithm used to assess the similarity between signals of varying lengths. Initially developed for automatic speech recognition, DTW has found applications in psycholinguistics, particularly in analyzing gesture form similarity. An open question in this domain is how effectively DTW captures gesture form similarity. Here, we validate DTW against human annotations of gesture form similarity across two multimodal interaction corpora and explore its utility as an automatic, continuous measure of gesture form similarity. Our findings reveal weak to moderate correlations between DTW distance and the number of similar gesture features – such as handshape, movement, orientation, and position – suggesting that DTW serves as a useful proxy for gesture form similarity. Additionally, we highlight the importance of qualitative analysis of raw data and DTW predictions in enhancing DTW’s predictive accuracy. Our study offers a rigorous validation of DTW as a measure of gesture form similarity and presents a detailed framework for preprocessing motion tracking data and calculating DTW distance. While none of the methods is perfect, the combination of automatic and manual measures provides a comprehensive approach to understanding and measuring gesture form similarity.

## Introduction

Over the past few decades, one of the key objectives in cognitive science has been to understand behavioral similarity, whether for understanding human behavior or for developing tools for automatic speech recognition. A widely used algorithm for quantifying the degree of similarity of two signals is *dynamic time warping* (DTW), which allows for comparing two sets of time series of different lengths and computes the minimum transformation needed (i.e., distance) to align the two signals (Müller, 2007). DTW was originally developed for speech recognition, but it has been applied in other areas, including data mining (Ratanamahatana & Keogh, 2004, 2005), economics (Raihan, 2017), and motion classification (Adistambha et al., 2008). Recently, psycholinguistic studies started applying DTW to quantify the degree of similarity in gestures (Pouw & Dixon, 2019; Pouw et al., 2021a, b; Trujillo et al., 2023). Yet, an open question in this domain is how DTW measures relate to manual annotations of gesture similarity. Therefore, the present study validates DTW with manual coding of gesture form similarity in two datasets and explores its utility as a measure of gesture form similarity.

Classic approaches to manual coding of gesture form similarity come with two major limitations: (i) manually assessing gesture form similarity is labor-intensive, and (ii) manual gesture form similarity coding is done in a binary fashion (i.e., similar or not similar), lacking details in the degree of gesture form similarity.

Typically, manual coding of gesture form similarity involves multiple researchers and research assistants evaluating the similarity of prime-target gesture pairs in a binary fashion, either holistically or per gesture feature (e.g., handshape, movement, orientation, position). This process can take up to months for a moderate sample size of 500 gesture pairs, including the time needed for training and establishing inter-rater reliability. This time-consuming process poses a challenge in measuring gesture form similarity.

Binary coding of gesture form similarity is widely used, presumably because it facilitates coding and statistical analyses. For example, deciding whether a pair of gestures is similar or not may be easier than deciding to what extent they are similar, on a scale of 1 to 5. Moreover, binary responses are one of the most dominant data types, and there are established methods to analyze them as dependent variables (e.g., using logistic regressions) or as independent variables (Brehm & Alday, 2022). However, binary coding of gesture form similarity forces the coder to decide whether a pair of gestures is similar or not, even though many cases fall between perfect similarity and no similarity (e.g., somewhat similar). This leads the gesture form similarity coding to miss the granularity of gesture form similarity.

Therefore, the present study explores dynamic time warping (DTW) as an alternative approach to address challenges in measuring gesture form similarity. DTW offers an automatic, continuous measure of gesture form similarity that may replace or complement manual coding.

### Applications of DTW in gesture studies

DTW was first applied to gestures in the field of computer vision for automatic gesture recognition purposes (e.g., Bautista et al., 2013; Celebi et al., 2013; Mezari & Maglogiannis, 2017; Reyes et al., 2011). Recently, psycholinguists have started using DTW to investigate the similarity in co-speech gestures (Pouw & Dixon, 2019; Pouw et al., 2021a; Trujillo et al., 2023) and silent gestures (Pouw et al., 2021a, b). In this section, we will summarize the kinematic variables used in these four psycholinguistic gesture studies and approaches to DTW validation to identify limitations.

In Pouw and Dixon (2019), the authors applied DTW to the 3D velocity of the dominant hand to construct a gesture network in which nodes with similar distance scores are placed closer to each other. Through gesture network analyses, they demonstrated that nodes for iconic gestures were further away from each other than those for beat gestures, suggesting that iconic gestures are more complex in their trajectories than beat gestures. Although the paper reported no explicit validation of DTW, the fact that the gesture network analysis distributed nodes for iconic gestures more sparsely than for beat gestures indicated that DTW is sensitive to gross distinctions in gesture type.

Unlike Pouw and Dixon (2019), in which DTW distance was computed based on velocity, the other studies computed distance based on 2D (*x*, *y*) or 3D (*x*, *y*, *z*) trajectories of wrists, thumbs, hand tips, index fingers, and/or head. In particular, Pouw et al. (2021b) used *z*-normalized 2D trajectories for left and right wrists, left and right index fingers, and the head. Pouw et al. (2021a) used *z*-normalized 3D trajectories for left and right wrists, left and right hand tips, and the head in Study 1, and *z*-normalized 3D trajectories for thumb and hand tip for right hand only in Study 2. In Trujillo et al. (2023), they first centered 2D trajectories for left and right wrists and the head relative to the initial neck position (i.e., *position normalization*) and computed DTW on the kinematic variables. It is worth noting that for hands, these studies use either wrists only or wrists with one additional keypoint for each hand (e.g., hand tip or index finger), which do not fully capture the similarity in hand orientation and handshape. Therefore, with the aim of capturing more fine-grained similarity of gestures, this study extends the previous research by using the wrists and the tips of all fingers for both hands.

DTW has been demonstrated to achieve high accuracy (from 75% to 96% average accuracy; see Dau et al., 2018 for details) in classifying motions, such as classifying (i) a set of 12 cricket umpire hand signs (Ko et al., 2005), (ii) Gun-Draw motions (i.e., drawing a replica gun from the holster, point it at a target, and return it to the holster) and Point motions (i.e., pointing with index fingers at a target; Dau et al., 2019), and (iii) eight different hand movement types (Liu et al., 2009). Moving beyond clear, isolated motions, Beecks et al. (2015) assessed the performance of DTW on 20 *co-speech gestures* involving either spiral (six), circular (eight), or straight (six) movements and showed that the DTW distances for the gestures belonging to the same movement type were lower than for those belonging to different movement types. Additionally, Pouw et al. (2021b) validated DTW by showing that DTW distance is reliably lower (more similar) for true gesture pairs compared to false random gesture pairs, which were created by randomly pairing two gestures that were neither in the same functional nor thematic category. Although previous studies have validated DTW on clear, isolated motions and on a small number of co-speech gestures, a rigorous validation of DTW is needed to explore its accuracy in measuring similarity in spontaneous gestures. Here, we aim to achieve this by examining the extent to which the DTW distance correlates with human ratings of gesture form similarity for spontaneously produced co-speech gestures.

### Present study

This study aims to explore the utility of DTW as a measure of gesture form similarity. To do so, we examine DTW’s accuracy in estimating gesture form similarity by taking human manual coding of gesture form similarity as “ground truth”^1^ and testing whether the DTW distance is lower for gestures manually annotated as similar. We perform such DTW validation on two sets of multimodal corpora of interactions from Rasenberg et al. (2022) (Study 1) and Akamine et al. (2025) (Study 2) in which a pair of participants freely used speech and gestures to repeatedly communicate about 16 images of novel objects over six consecutive rounds.

For each dataset, we examine the association between DTW and manual coding at two levels: overall similarity and feature-level similarity. Our primary analysis focuses on the association between DTW and the overall similarity, which is calculated by counting the number of features annotated as similar. If DTW captures gesture form similarity, we expect a negative association between the DTW distance and the overall gesture form similarity, such that the DTW distance is low when the overall gesture similarity is high. In Study 2, we also perform qualitative checks on incongruent cases where DTW indicates high gesture similarity while manual coding indicates low gesture similarity or vice versa. This allows us to identify the differences between DTW and manual similarity coding and areas for improvement. Feature-level analyses follow the overall similarity analyses to explore in which feature(s) DTW can capture similarity.

## General approach

To prepare time series data for DTW, we extracted 3D keypoint positions using MediaPipe (Lugaresi et al., 2019; Pouw & Akamine, 2025). Python scripts for keypoint extraction using MediaPipe, preprocessing, and DTW computation are available at https://github.com/ShoAkamine/dtw_validation.

**Fig. 1:**
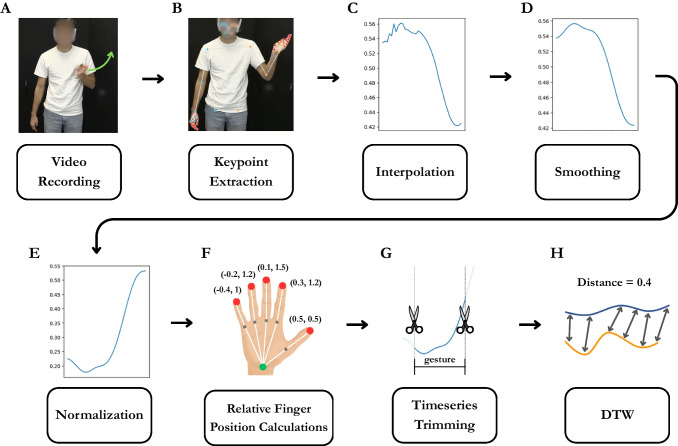
Time series data processing workflow. **A** An example gesture, and **B–E** results of keypoint extraction, interpolation, smoothing, and normalization for the gesture. **F** Relative finger position calculations for the *x*- and *y*-axes (*x*, *y*). Values for relative finger positions are increased for illustrative purposes; actual values are much smaller. **G** Time series data trimming, by which time series data for each target iconic gesture were extracted based on manual gesture annotations. **H** Dynamic time warping (DTW) and an example DTW distance

### Preprocessing

To prepare time series data for DTW, we first extracted 3D keypoint positions using MediaPipe, a video-based motion tracking pipeline developed by Google (Lugaresi et al., 2019). We adapted the script openly shared on the EnvisionBOX website for running MediaPipe and storing the motion tracking time series data (Pouw & Akamine, 2025). The output CSV files contained the standardized location (0–1) of each keypoint for the *x*-, *y*-, and *z*-axes per frame. As MediaPipe is a non-invasive, video-based motion tracking system, it often fails to estimate the position of visually occluded keypoints. Because DTW returns errors if the dataframe contains empty rows, we filled in missing values with a linear interpolation, such that a vector of [1, 2, NA, NA, 5] becomes [1, 2, 3, 4, 5].

After interpolation, we smoothed the time series data by applying a Gaussian filter (sigma = 2) to reduce the influence of jitter and false estimation on the accuracy of DTW estimation (see Fig. 1D). Then, we performed a set of normalizations, namely position normalization, size normalization, aspect ratio adjustment, and *y*-axis inversion. Position normalization was performed to minimize the influence of differences in standing positions on the accuracy of DTW estimation. For example, if one participant is positioned on the left side of the video and the other participant is positioned on the right, the DTW distance will increase due to the different standing positions. To tackle this issue, we centered all the keypoints so that the position of the middle torso always becomes [*x* = 0, *y* = 0, *z* = 0] in each frame, and the positions of the other keypoints become relative to the mid-torso. This means that all keypoints to the left of mid-torso (from an observer perspective) will have negative values in the *x*-axis, and those to the right will have positive values. Likewise, keypoints located above the mid-torso will have *negative* *y* values and those located below will have *positive* *y* values (*y*-axis will be inverted later to make the interpretation more intuitive). We also normalized the data for size to account for differences in height and proximity to the camera. In particular, we calculated a scale factor as the Euclidean distance in 3D space between the mid-shoulder and mid-hip, and multiplied each keypoint by the scale factor. It is worth noting that although one can achieve position and size normalization by *z*-normalizing the data (e.g., Dau et al., 2019; Pouw et al., 2021b; Rakthanmanon et al., 2013; Wu et al., 2021), the predictive accuracy of DTW was lower for *z*-normalized data compared to data normalized based on our approach. Next, we multiplied all *x*-axis values by 1.78 (16/9) to adjust for the aspect ratio of 16:9 (e.g., 1980 x 1080 resolution). This was necessary to align the units of the *x*-axis and *y*-axis. For example, without aspect ratio adjustment, if moving a hand 5 cm horizontally corresponds to 0.1 in *x*, moving a hand 5 cm vertically corresponds to 0.178 in *y* because the range of the MediaPipe unit is 0 to 1 for both *x* and *y*, despite the width of the video being 1.78 times larger than the height. This issue can be resolved by multiplying the *x* values by 1.78. Lastly, we inverted the *y*-axis so that higher values indicate higher in space. The outcome of the normalizations can be seen by comparing the plots for smoothing (D) and normalization (E) in Fig. 1.

Next, we calculated the position of each fingertip relative to the wrist as the displacement (distance and direction) between the wrist and the fingertip by subtracting the coordinates of each hand’s wrist from the coordinates of the corresponding hand’s fingertips per frame. The resulting *relative finger positions* encode spatial configurations of fingertips relative to the wrist and are agnostic to differences in hand position. For example, if the coordinates for the right wrist are [*x* = 1, *y* = 1] and for the index fingertip [*x* = 1.3, *y* = 2.2], the relative position of the index fingertip will be [*x* = 0.3, *y* = 1.2], indicating that the index fingertip is located upper right to the wrist. On the other hand, if the position of the right wrist is different [*x* = 2, *y* = 2] but the spatial relation between the wrist and index fingertips remains identical [*x* = 2.3, *y* = 3.2], the relative index finger position will be identical too [*x* = 0.3, *y* = 1.2]. This demonstrates that relative finger positions maintain information about the spatial relationship between the wrist and fingertips while ignoring differences in hand position. Therefore, we used relative finger positions instead of absolute finger positions to avoid inflating the effect of misalignment in hand position. By using relative finger positions, the information about hand position will be encoded only in the keypoints for the wrists, while the information about handshape and orientation should be encoded in the keypoints for the fingertips. For a visual illustration of relative finger positions, see Fig. 1F.

Lastly, the preprocessed time series data were then merged with ELAN annotations to extract the time series data for each target iconic gesture pair. To ensure that the time series is long enough to produce reliable distance scores, any gestures whose duration was shorter than 330 ms (ten frames in 30 FPS) were lengthened to 330 ms by adding equal intervals before and after the annotated strokes. In Study 1, 265 gesture pairs (65.3%) consisted of at least one iconic gesture whose duration was shorter than 330 ms, and Study 2 contained 55 such cases among 100 gesture pairs. The full preprocessing workflow is illustrated in Fig. 1.

**Fig. 2:**
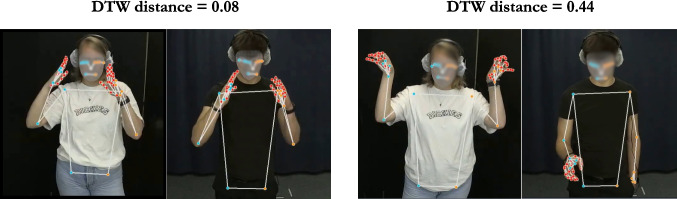
Example similar and dissimilar pairs of referentially aligned iconic gestures. The *left figure* shows the normalized DTW distance for a similar gesture pair, and the *right figure* shows the normalized DTW distance for a less similar gesture pair

### DTW

To measure the similarity of pairs of referentially aligned gestures, we computed the DTW distance for each aligned gesture pair by using an asymmetric, open-begin-end DTW implemented in the dtw-python package (version 1.5.1; Giorgino (2009); Tormene et al. (2009)). We used open-begin-end DTW because segmenting gesture onsets and offsets is challenging, and suboptimal segmentation degrades DTW accuracy (Silva et al., 2016; Tormene et al., 2009). This variant of DTW mitigates the issue by allowing portions at the start and end of the time series to be ignored. The DTW distance was computed separately for each hand’s wrist and fingertips, then averaged across these six keypoints to obtain one distance value for each hand. Because DTW distance is accumulative and thus will be larger for gestures with longer duration, we normalized the distance by dividing the cumulative distance by the total number of frames for two gestures (Giorgino, 2009; Tormene et al., 2009; Characteristics of original and normalized DTW distance are illustrated in the “dtw_simulation.ipynb” file available at https://github.com/ShoAkamine/dtw_validation under “kinematics/simulation”). To improve the accuracy of the distance scores, we averaged the distance only for gesturing hands. For example, in the case of two speakers producing left-handed gestures, we used the distance for the left-hand keypoints and calculated the mean distance. If speaker A produced a left-handed gesture and speaker B produced a right-handed gesture, then we computed the distance between A’s left hand and B’s right hand. Figure 2 shows the normalized DTW distance for a similar and less similar aligned gesture pair.

## Study 1

In this study, we validate DTW with manual annotations of gesture form similarity for the multimodal corpus of interaction used in Rasenberg et al. (2022). We use this dataset because DTW has been applied to Kinect-collected motion tracking data from this dataset in Pouw et al. (2021a), and it provides not only audio/video recordings but also manual annotations of gestures and gesture form similarity. In the experiment, 19 pairs of participants alternated roles as director and matcher, freely communicating about images of 16 novel objects in a face-to-face setting. The corpus provides video recordings, speech, and gesture annotations as ELAN files (Wittenburg et al., 2006), and manual annotation of gesture form similarity. We prepared the motion-tracking time series data by following the procedure described in the “General approach” section.

**Fig. 3:**
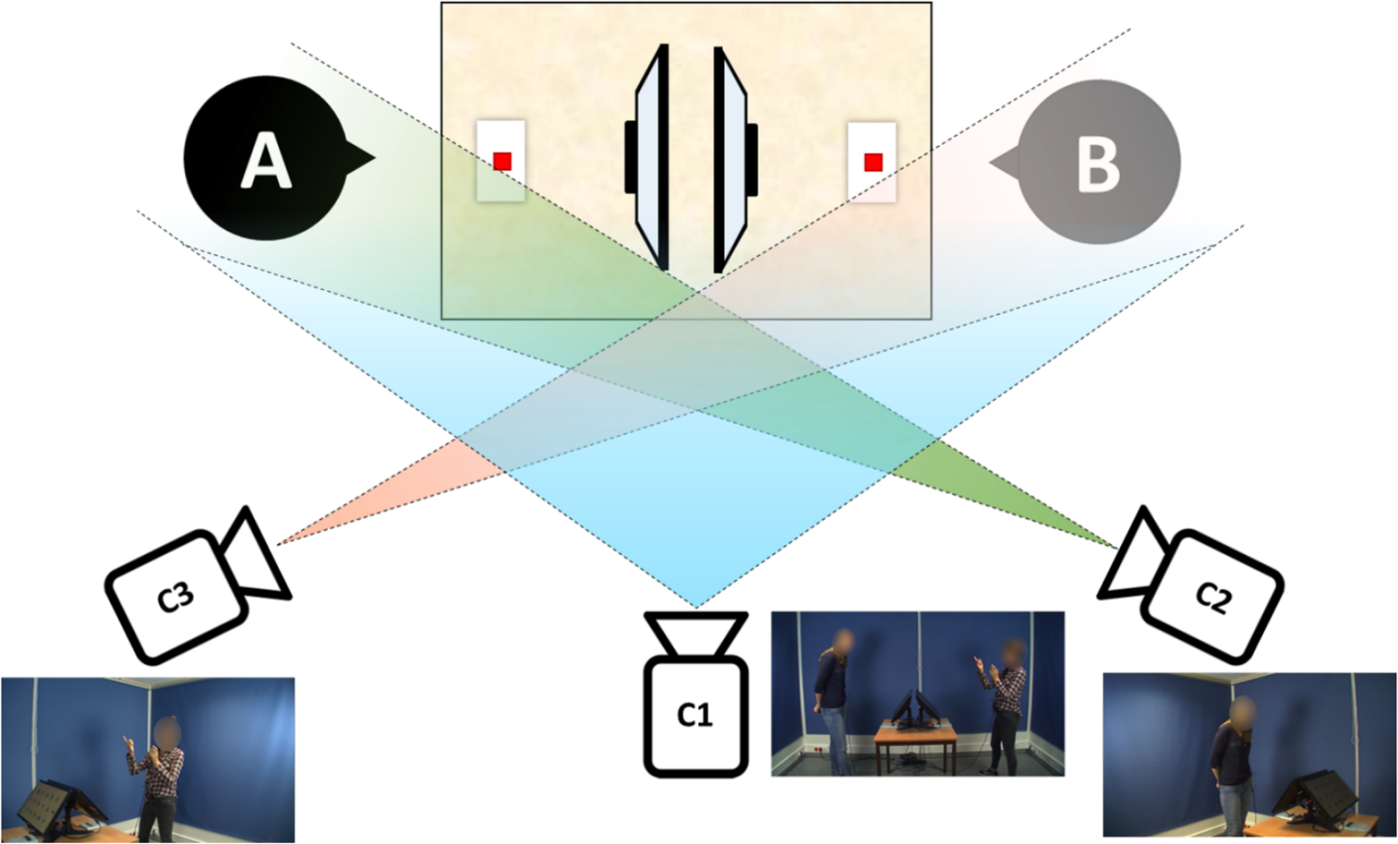
Visual illustration of the recording setup in Rasenberg et al. (2022). Video recordings from C2 and C3 were used for DTW analyses

Interaction was recorded using three HD cameras (JVC GY-HM100/150). The frame rate was set to 30 FPS; 1 s of recording consists of 30 images. One camera was positioned on the side of the participants to capture both participants simultaneously. The other cameras were used to record each participant separately: Participant A was recorded diagonally from their front-right side, and Participant B was recorded diagonally from their front-left side (see Fig. 3). For DTW analyses, the video recordings for each participant were used.

The similarity in gesture form was annotated by a trained assistant for a subset of gestures (for eight dyads in round 1 and round 2, *n* = 406 gesture pairs) in terms of five features: handedness, handshape, movement, orientation, and position. Handedness was coded as 1 if two speakers gestured with the same hands, such as both speakers producing two-handed gestures, left-handed gestures, or right-handed gestures. If they used different hands when gesturing (e.g., speaker A produced a left-handed gesture, and speaker B produced a two-handed gesture), the handedness was coded as 0. For other features, 1 was assigned when two gestures were overall similar, and 0 was assigned otherwise. The coding was done without any contextual information: coders had access to neither the co-occurring speech nor the gesture referent. Inter-rater reliability was assessed for each feature based on a subset of data (*n* = 103 gesture pairs) and was established for handshape (agreement = 88.3%, Cohen’s kappa = .71), movement (agreement = 85.4%, Cohen’s kappa = .63), and orientation (agreement = 75.7%, Cohen’s kappa = .54). Although the chance-corrected Cohen’s kappa score was not higher than the threshold of 0.5 for position (agreement = 77.7%, Cohen’s kappa = .47), we included the coding for position in the subsequent analyses given that the raw agreement score was high. For more details on gesture form similarity coding and the inter-rater reliability procedure, see Appendix A in Rasenberg (2023).

We computed a Spearman’s rank correlation coefficient between the normalized DTW distance scores and the manual similarity rating scores to examine whether the distance was smaller for more similar gestures. In particular, we examined whether there was a negative correlation between the DTW distance and the number of features annotated as similar for each gesture pair, which was calculated by summing the similarity ratings for handshape, movement, orientation, and position. For example, if a pair of gestures were rated as similar in terms of handshape and position, the number of overlapping features would be 2. We excluded handedness because we computed the DTW distance for gesturing hands only. To investigate the relation between the DTW distance and manual similarity rating in more detail while controlling for differences in the overall mean DTW distance across participants and items, we modeled the normalized DTW distance scores as a function of the manual similarity ratings using linear mixed-effects regression models with by-dyad and by-item varying intercepts. Besides inspecting the association between the DTW distance and the number of overlapping features, we examined for each feature separately whether the DTW distance was smaller for similar gesture pairs. Lastly, to determine which features had a stronger influence on the DTW distance, we included the similarity rating for each feature as fixed effects in a linear mixed-effects model to inspect the effect size of each feature *when controlling for the other features*. All statistical analyses were conducted in R (Version 4.4.0 R Core Team, 2024), and the *lme4* package (Version 1.1-35.3; Bates et al., 2015 was used for linear mixed-effects models. R markdown files and processed data used for statistical analyses are available at https://github.com/ShoAkamine/dtw_validation.

**Fig. 4:**
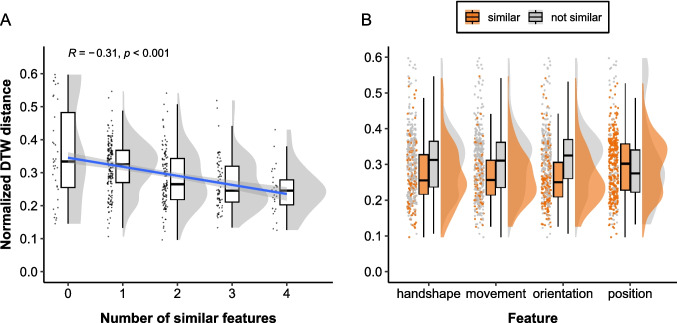
Normalized DTW distance by manual annotation of gesture form similarity. Each *dot* represents a pair of iconic gestures. **A** Relation between the normalized DTW distance and the number of features annotated as similar. The regression line from a linear model is shown as a *blue line* with its 95% confidence interval in the shared area. **B** Distribution of DTW distance for gesture pairs annotated as similar and not similar per feature

### Results

A Spearman’s rank correlation coefficient indicated a weak negative correlation between the DTW distance and the number of features manually annotated as similar ($r_s(404) = -.311$, $p <.001$), indicating that the DTW distance is smaller for pairs of gestures that align in more features, as illustrated in Fig. 4A. A linear mixed-effects regression model with random intercepts for dyads and items revealed a similar pattern ($\beta = -0.02$, $SE =.004$, $t = -5.19$, $p <.001$), indicating that the negative association between the DTW distance and the number of similar features holds when accounting for differences in mean across dyads and items.

To investigate in which features the DTW distance is negatively associated with manual gesture form similarity coding, we run a set of linear mixed-effects models, each including one of the four features as fixed effects. We found significant negative slopes for handshape ($\beta = -0.027$, $SE =.009$, $t = -3.09$, $p <.01$), movement ($\beta = -0.04$, $SE =.01$, $t = -4.17$, $p <.001$), orientation ($\beta = -0.046$, $SE =.008$, $t = -5.71$, $p <.001$). The estimated slope for position was not significant ($\beta = 0.016$, $SE =.01$, $t = 1.53$, $p =.13$). See Fig. 4B for the distribution of DTW distance scores.

To determine which features have a stronger influence on the DTW distance, we performed a linear mixed-effects model with the similarity coding for handshape, movement, orientation, and position as separate fixed effects. The model showed that the normalized DTW distance is significantly lower when two gestures are annotated as similar in terms of movement ($\beta = -0.03$, $SE =.009$, $t = -3.17$, $p <.01$) and orientation ($\beta = -0.038$, $SE =.009$, $t = -4.45$, $p <.001$), but not in handshape ($\beta = -0.011$, $SE =.009$, $t = -1.19$, $p =.24$) and position ($\beta = 0.016$, $SE =.01$, $t = 1.67$, $p =.10$) when controlling for the other three features. The correlation of fixed effects revealed a weak negative correlation between handshape and orientation ($- 0.3$), suggesting that when the slope for orientation is more extremely negative, the slope for handshape becomes flatter. This is likely because handshape and orientation are both reflected in the relative positions of the finger tips.

### Discussion

In this study, we aimed to validate DTW with manual annotation of gesture form similarity to explore its utility as a measure of gesture form similarity. We found a significant negative association between the DTW distance and the number of features annotated as similar, such that similar gesture pairs were associated with lower DTW distance. Furthermore, there was a significant negative association between the DTW distance and the manual similarity rating in all four features except position. These results suggest that DTW can capture overall gesture form similarity, and thus, the DTW distance can serve as a measure of gesture form similarity.

A more detailed feature-based analysis demonstrated that movement and orientation are significant predictors of the DTW distance, with the magnitude of effect size being largest for orientation. The handshape and position were not significant predictors of the DTW distance when controlling for the other three features. This suggests that in this dataset, the DTW distance is primarily influenced by orientation and movement, and differences in handshape and position did not affect the DTW distance.

It is important to note that the video recordings were not optimized for motion tracking. As shown in Fig. 3, each participant was recorded from different angles. This means that the identical handshapes and movements may look different in the videos due to different camera angles, introducing noise in the data that may affect the accuracy of DTW. In addition, the camera settings, such as frame rate and shutter speed, were not optimized for kinematic analyses either, resulting in blurred hands for fast movements and, hence, less accurate pose estimation. To tackle these issues, we validate DTW again using video recordings better suited for kinematic detection in Study 2.

## Study 2

Study 1 demonstrated that DTW effectively captures overall gesture form similarity, as well as similarity in movement and orientation. To test whether the results of Study 1 can be generalized to different datasets, we validate DTW on another multimodal corpus of interaction (Akamine et al., 2025), in which 45 pairs of Dutch-speaking participants completed the same referential communication task as in Study 1, but on Zoom. The corpus provides video recordings and ELAN files for speech and gesture annotations. The motion tracking time series data were generated by following the procedure described in the “General approach” section. Video recordings were optimized for kinematic analyses by having each participant recorded from the front with a higher frame rate (50 FPS) and shutter speed (1/250). In addition, we attempt to rate the gesture form similarity in a more nuanced way: instead of binary coding for each feature, we rated gesture form similarity on a scale from 0–5 in terms of handshape, movement, orientation, and position to capture the degree of similarity in gesture form. For the main analyses, we converted the similarity rating to binary data (i.e., similar and not similar) to ensure consistency in gesture form similarity rating across the studies. The analysis with the original scale of gesture form similarity rating can be found in Appendix A.

In the experiment, participants performed the referential communication task on Zoom, each joining the Zoom meeting from a different room. Each room was equipped with two 50 x 50 cm or 40 x 60 cm front lights and a Canon XF205 video camera with a Sennheiser ME64 cardioid condenser microphone.

The similarity in gesture form was annotated by a trained assistant for a subset of randomly selected referentially aligned pairs of iconic gestures (*n* = 100 gesture pairs) in terms of handshape, movement, orientation, and position. The degree of similarity was evaluated on a scale of 0–5, 0 indicating no similarity and 5 indicating perfect similarity. For the main analyses, we converted the similarity rating to binary data: scores of 4 and 5 were recoded as similar, and scores of 0–3 were recoded as not similar. The criteria for binary categorization of similarity were set in such a way that the ratio of similar and not similar ratings is consistent across two corpora (0.752 for the corpus used in Study 1 and 0.754 for the corpus used in Study 2). For handshape, we rated the similarity in overall handshape and the change in handshape. For example, if both speakers produced a C-shaped handshape and moved the fingers up and down to depict Pac-Man, we assigned a high similarity rating for the gesture pair. On the other hand, if the speakers produced the same C-shaped handshape but one gesture made up-and-down movements, and the other did not, we assigned a moderate similarity rating. If the gestures overlapped neither in overall handshape nor in handshape change, we assigned a low similarity rating. Similarly, we rated the similarity in orientation in terms of overall orientation and change in orientation. As for movement, we rated wrist movement similarity based on trajectory shape and direction. Only when gesture pairs were aligned in trajectory shape and movement direction was a high similarity score given. When they were aligned in either trajectory shape or movement direction, we assigned a moderate similarity rating. When neither the trajectory shape nor the movement was aligned, we assigned a low similarity rating. Lastly, the similarity in hand position was rated in terms of horizontal (*x*), vertical (*y*), and depth (*z*) axes. For example, if one gesture was produced on the top left and the other on the middle left, a moderate similarity rating was assigned because they were aligned on the *x*-axis but not on the *y*-axis. On the other hand, if a gesture was produced on the top left and the other on the bottom right, we assigned a low similarity rating. As in Study 1, the coder did not have access to any information about co-occurring speech, gesture referent, or trial information.

We computed a Spearman’s rank correlation coefficient between the normalized DTW distance scores and the number of features manually annotated as similar to examine whether the DTW distance was smaller for more similar gestures. To investigate the relation between the DTW distance and manual similarity ratings more in detail while controlling for differences in the mean DTW distance across dyads and items, we performed linear mixed-effects regression models on the DTW distance as a function of the number of similar features with by-dyad and by-item varying intercepts. We also examined, for each feature separately, whether the DTW distance was smaller for similar gesture pairs. Lastly, to determine which features had a stronger influence on the DTW distance, we included the similarity rating for each feature as a separate fixed effect in a linear mixed-effects model to inspect the effect size of each feature when controlling for the other features.

**Fig. 5:**
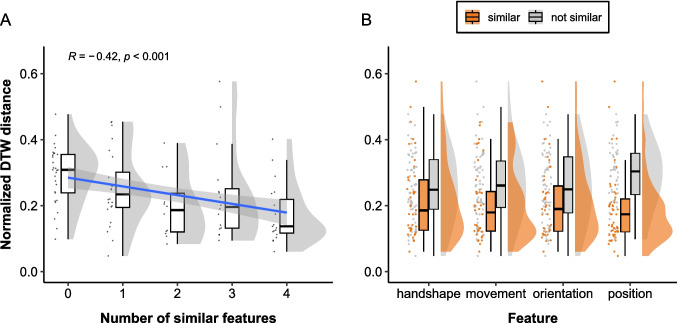
Normalized DTW distance by manual annotation of gesture form similarity. Each *dot* represents a pair of iconic gestures. **A** Relation between the normalized DTW distance and the number of features annotated as similar. The regression line from a linear model is shown as a *blue line* with its 95% confidence interval in the shared area. **B** Relation between the normalized DTW distance and gesture form similarity rating per feature

### Results

A Spearman’s rank correlation coefficient revealed a moderate negative correlation between the DTW distance and the number of features rated as similar ($r_s (98) = -.42$, $p <.001$), indicating that the DTW distance is smaller for gesture pairs that are rated as more similar, as illustrated in Fig. 5A. A linear mixed-effects regression model with random intercepts for dyads and items also revealed a significant negative slope ($\beta = -0.027$, $SE =.007$, $t = -3.80$, $p <.001$), indicating that the negative association between the DTW distance and the number of similar features holds when accounting for differences in mean across dyads and items.

Another set of linear mixed-effects models, each including the binary similarity rating for one of the four features as fixed effects, demonstrated significant negative slopes for movement ($\beta = -0.062$, $SE = 0.022$, $t = -2.79$, $p <.01$) and position ($\beta = -0.103$, $SE = 0.02$, $t = -5.17$, $p <.001$). Although negative associations were also observed for handshape and orientation, they were not significant (handshape: $\beta = -0.036$, $SE = 0.023$, $t = -1.60$, $p =.11$; orientation: $\beta = -0.043$, $SE = 0.023$, $t = -1.92$, $p =.06$). See Fig. 5B for the distribution of DTW distance scores per feature.

We also performed a linear mixed-effects model on the manual similarity rating for all four features as separate fixed effects to determine which features have a stronger influence on the DTW distance. The model showed that the normalized DTW distance is significantly lower for gesture pairs rated as highly similar in terms of position only ($\beta = -0.095$, $SE = 0.022$, $t = -4.27$, $p <.001$), but not in the other features (handshape: $\beta = 0.007$, $SE = 0.027$, $t = 0.27$, $p =.79$; movement: $\beta = -0.029$, $SE = 0.025$, $t = -1.18$, $p =.24$; orientation: $\beta = 0.001$, $SE = 0.028$, $t = 0.03$, $p =.98$), when controlling for the other three features. The correlation of fixed effects revealed a moderate negative correlation between handshape and orientation ($-0.50$), suggesting that when the negative slope for orientation is steeper, the slope for handshape becomes flatter. The negative correlation in the fixed effects may stem from the fact that handshape and orientation are reflected in the relative positions of the finger tips.

**Fig. 6:**
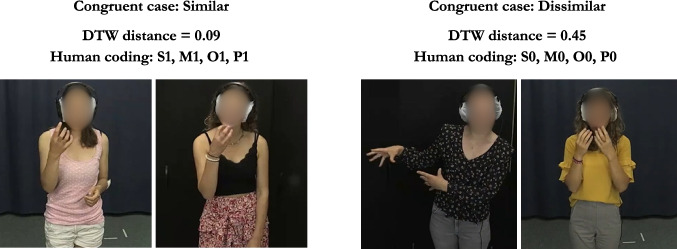
Example similar or dissimilar gesture pairs for which human ratings and DTW prediction were congruent. The *left figure* shows an example similar gesture pair. The gesture pair was rated by humans as similar in all four features (i.e., handshape, movement, orientation, and position), and the DTW distance was low. The *right figure* shows an example dissimilar gesture pair, which was rated as not similar in all four features, and its DTW distance was high

### Discussion

In this study, we used another dataset more optimized for kinematic analyses to validate DTW with human ratings of gesture form similarity to investigate whether the DTW’s utility demonstrated in Study 1 can be generalized to other datasets. We found a significant negative association between the DTW distance and the number of similar features, and the same pattern was observed when accounting for differences in mean DTW distance score across dyads and items. Furthermore, there was a significant negative association between the DTW distance and the manual similarity rating for movement and position. These results show that DTW can capture gesture form similarity in overall similarity and in movement and position.

A feature-level analysis demonstrated that position was the only significant predictor of the DTW distance when controlling for the other three features. Inspecting the correlation of fixed effects revealed a moderate negative correlation between handshape and orientation, presumably because the information about handshape and orientation is reflected in the relative positions of finger tips. This calls for developing a method that quantifies similarity for each feature independently, if per-feature similarity is of interest.

It is worth noting that although we observed a significant negative association between the DTW distance and human rating of gesture form similarity, the strength of the correlation was weak in Study 1 ($r_s = -.311$) and moderate in Study 2 ($r_s =-.42$). To understand the causes of relatively weak correlation, we qualitatively examine the data from Study 2 and seek ways to improve the accuracy of the DTW estimation.

## Qualitative validation

To explore why the correlation between the DTW distance and the gesture form similarity rating was relatively weak, we qualitatively examined cases where human coders and DTW’s predictions were congruent (see Fig. 6), and most importantly, incongruent cases where human coders rated the gesture pairs as highly similar while the DTW distance predicted otherwise, or vice versa. The qualitative inspection of data from Study 2 revealed two major causes of incongruence: (i) the production of mirrored gestures, and (ii) debatable coding decisions.

**Fig. 7:**
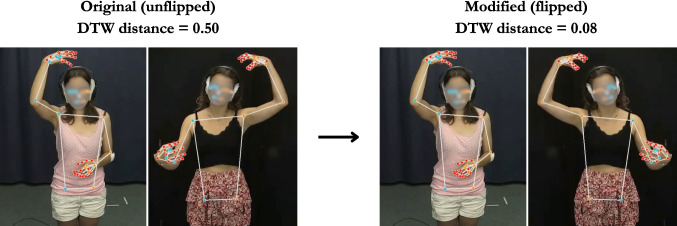
Example mirrored gesture pair in which participant A produced a right-handed “wearing-a-hat” gesture and participant B produced the same gesture but in the opposite hand. A human annotator rated it as highly similar: The number of aligned features was three (handshape, orientation, and position). In the original version, the normalized DTW distance was extremely high. After flipping the video for participant B, the DTW distance reduced significantly, being aligned more with the human rating

Among seven incongruent cases where the human annotator rated the gesture pair to be highly similar while the DTW distance was high, five of them involved the production of mirrored gestures, in which one participant produced a gesture using their left hand and the other participant produced a similar gesture using their right hand. Although humans can easily perceive similarity in such mirrored gestures, the current approach fails to adapt to these cases, resulting in misalignment between human gesture form similarity ratings and the DTW distance. To overcome this issue, we modified the pipeline in such a way that when participants produced one-handed gestures but in different hands (e.g., A producing a left-hand gesture and B producing a right-hand gesture), DTW compares participant A’s gesture with participant B’s original gesture and flipped gesture and takes the one with the lower distance. For example, if the distance for the original gesture pair is 0.5 while the distance between A’s gesture and B’s flipped gesture is 0.1, we adopt the latter. Such *x*-axis inversion has been applied in other studies as well (e.g., Pearson et al., 2024). A qualitative inspection revealed that the modified approach can successfully capture gesture form similarity in mirrored gesture production, as illustrated in Fig. 7.

**Fig. 8:**
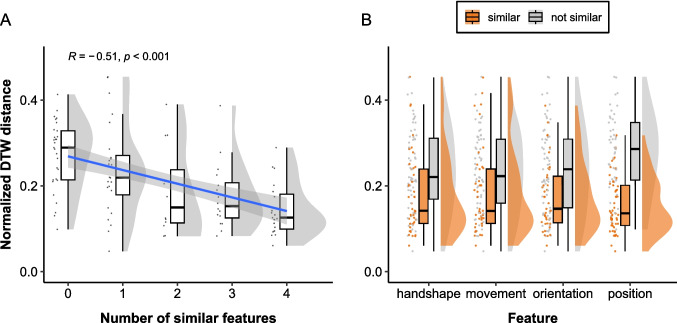
Normalized DTW distance for the modified pipeline by manual annotation of gesture form similarity. Each *dot* represents a pair of iconic gestures. **A** Relation between the normalized DTW distance and the number of features annotated as similar. The regression line from a linear model is shown as a *blue line* with its 95% confidence interval in the shared area. **B** Relation between the normalized DTW distance and gesture form similarity rating per feature

To assess whether the modified approach improves DTW estimation accuracy, we performed the same analyses on the DTW distance scores generated by the modified pipeline. A Spearman’s rank correlation coefficient revealed a moderate negative correlation between the DTW distance and the number of features rated as similar ($r_s (98) = -.51$, $p <.001$), which is a stronger negative correlation than the one reported in Study 2 ($r_s = -.42$, difference = .09). A linear mixed-effects regression model with random intercepts for dyads and items also revealed a significant negative slope ($\beta = -0.032$^2^, $SE =.006$, $t = -5.58$, $p <.001$), indicating that the negative association between the DTW distance and the number of similar features holds when accounting for differences in mean across dyads and items (see Fig. 8A).

Another set of linear mixed-effects models, each including the binary similarity rating for one of the four features as fixed effects, demonstrated significant negative slopes for all features (handshape: $\beta = -0.06$, $SE = 0.019$, $t = -3.17$, $p <.01$; movement: $\beta = -0.048$, $SE = 0.019$, $t = -2.47$, $p <.05$; orientation: $\beta = -0.066$, $SE = 0.019$, $t = -3.47$, $p <.001$; position: $\beta = -0.122$, $SE = 0.016$, $t = -7.90$, $p <.001$). See Fig. 8B for the distribution of DTW distance scores per feature.

We also performed a linear mixed-effects model on the manual similarity rating for all four features as separate fixed effects to determine which features have a stronger influence on the DTW distance. The model showed that the normalized DTW distance is significantly lower for gesture pairs rated as highly similar in terms of position only ($\beta = -0.116$, $SE = 0.017$, $t = -6.72$, $p <.001$), but not in the other features (handshape: $\beta = -0.02$, $SE = 0.021$, $t = -0.94$, $p =.35$; movement: $\beta = 0.014$, $SE = 0.019$, $t = 0.74$, $p =.46$; orientation: $\beta = -0.016$, $SE = 0.021$, $t = -0.77$, $p =.44$), when controlling for the other three features. The fixed-effect correlation revealed a moderate negative association between handshape and orientation ($-0.50$).

Misalignment between the similarity coding and the DTW distance in other incongruent cases, especially when manual gesture form similarity coding indicated low similarity while DTW predicted otherwise, also revealed some coding decisions on which some of the authors disagreed with the annotator. This suggests that DTW may also be useful in detecting potential coding discrepancies.

## General discussion

Using two multimodal corpora of interaction, we have demonstrated that dynamic time warping (DTW) distance negatively correlates with human coding of gesture form similarity ($r_s$ ranging from -.311 to -.51), indicating that the DTW distance is lower for gesture pairs that were annotated as similar by human coders. Feature-level analyses revealed significantly negative slopes for handshape, movement, and orientation in Study 1 and for all features in the exploratory analysis, in which we applied a modified DTW computation pipeline on the dataset used in Study 2. These findings demonstrate that DTW effectively captures gesture form similarity for spontaneous co-speech gestures. Unlike manual coding, which is labor-intensive and may be coarse-grained or categorical, DTW offers a time-efficient and continuous measure of gesture form similarity, making it a suitable approach to measuring gesture form similarity. It is worth mentioning that although automatic approaches to measuring gesture form similarity, such as quantifying kinematic similarity using DTW, allow researchers to investigate the degree of gesture form similarity efficiently, we advise researchers to always perform manual coding of gesture form similarity on a subset of their dataset. This is because manual coding is among the best ways to understand the data and improve the accuracy of kinematic measures, as illustrated in our exploratory analysis. Also, validating kinematic measures of gesture form similarity with the manual coding for a subset of data ensures that these measures actually do capture similarity for their data.

Although our main interest is to investigate whether the DTW distance is negatively associated with the manual similarity coding in terms of overall similarity as well as per feature, we also investigated which features have a stronger influence on the DTW distance in each corpus. Linear mixed-effects models, including the manual coding of each feature included as separate fixed effects, showed that the DTW distance was mostly affected by orientation and moderately by movement in Study 1, while in Study 2 and exploratory analysis, it was mostly affected by position only. Although the factors causing differences in each feature’s size of influence on the DTW distance across the datasets are unclear, we speculate that they stem from differences in recording setups and sample sizes. Participants were captured from angled positions in Study 1, while they were captured from their front in Study 2. Because of this, the difference in the wrist positions on the *x*-axis is less prominent in angled video recordings than in those recorded from the front, leading to a smaller influence of position on the DTW distance in Study 1. In addition, the estimates for handshape, movement, and orientation may not have been significant because of a smaller sample size of Study 2 (*n* = 100 comparisons) compared to Study 1 (*n* = 406 comparisons). Regardless of the causes, it is important to bear in mind which features have a stronger influence on the DTW distance for the dataset of interest when interpreting the results.

A qualitative inspection revealed a noteworthy difference between human raters and DTW: human coders rated mirrored gesture pairs as similar, whereas DTW predicted them as highly dissimilar in the original approach. To align DTW’s prediction more with manual coding, for pairs of one-handed gestures produced in opposite hands by different speakers (e.g., speaker A producing a left-handed gesture and speaker B producing a right-handed gesture), we flipped the video for participant B, computed two distance scores, one between the original, unflipped videos for participant A and B and the other between the participant A’s original video and B’s flipped video, and chose the one with a lower distance score. The modified approach increased the strength of correlation by .09, from $-.42$ to $-.51$, indicating a better alignment with the human gesture form similarity coding than with the original approach. This suggests that although humans can naturally perceive similarity in mirrored gesture pairs, kinematic similarity measures require an additional algorithm to detect similarity in such mirrored gesture pairs. This also emphasizes the importance of identifying systematic differences between DTW and humans via qualitative checks of incongruent cases.

### Limitations

The most obvious limitation of DTW is that the correlations between human ratings of gesture form similarity and DTW distance were moderate at best. Assuming that the human ratings constitute the gold standard, this implies that DTW is a useful, but rough proxy for perceived similarity. In addition, unlike manual coding, DTW cannot classify similarity per feature. These limitations suggest the complementary nature of these methods: human coding is costly and categorical but can classify gesture form similarity per feature (e.g., movement trajectory, handshape), whereas DTW can efficiently quantify gesture form similarity but cannot classify similarity per feature. Therefore, we recommend that researchers consider the strengths and limitations of each method and decide on the measure(s) that are suitable for answering the research questions. For example, if one is interested in investigating which phonological features sign language learners struggle to acquire, manual coding is a better choice because DTW cannot classify which features of the learner’s sign (e.g., handshape, movement, orientation) are similar to the target sign. In contrast, if one is interested in assessing the level of sign language vocabulary learning by using the similarity between learners’ signs and target signs produced by native signers as a proxy, DTW may be a better choice because it is time-efficient and provides an objective, continuous similarity measure that does not raise reliability issues as DTW returns identical results when applied multiple times on the same data.

The third limitation is that while our approach can automate the assessment of overall gesture form similarity, it still requires manual segmentation of gestures accompanying speech. Recent development in computational approaches to the multimodal language production exhibits a promising progress in automatic gesture detection (Ghaleb et al., 2024; Pouw et al., 2025). By combining our DTW pipeline with these automatic co-speech gesture detection models, these manual processes can be greatly automated. As completing gesture segmentation and gesture form similarity coding can each take several months, if not years, this increases the efficiency in analyzing gesture form similarity significantly. However, we recommend that researchers always perform manual coding for a subset of data and validate DTW with the manual coding. This way, they can ensure that DTW captures gesture form similarity for their data and detect cases where DTW fails to capture similarity, through which one can modify the DTW pipeline for improved predictive accuracy, as we did in the qualitative validation.

### Potential applications

One important area in which DTW can be useful is research on gestural alignment. People coordinate actions and become in tune with others in social interactions. Such *behavioral alignment* has been studied in non-communicative bodily actions (e.g., foot shaking; Chartrand & Bargh, 1999; Lakin & Chartrand, 2003) and in communicative behaviors such as spoken language use (e.g., Branigan et al., 2000; Brennan & Clark, 1996; Levelt & Kelter, 1982) and in co-speech gestures (e.g., Bergmann & Kopp, 2012; Holler & Wilkin, 2011; Kimbara, 2006; Kopp & Bergmann, 2013; Oben & Brône, 2016; Rasenberg et al., 2022). For example, Akamine et al. (2024) demonstrated that speakers repeatedly mimic each other’s word choices and gestures throughout a repeated reference game. By using our DTW pipeline, one can go beyond the frequency of gestural alignment and explore the degree of alignment in gesture form, which informs us more about the nature and functions of gestural alignment in social interaction.

DTW can also be applied to sign language research. In a recent study, Karadöller et al. (2024) investigated whether non-signing, hearing speakers use iconic-form mappings in signs or their own gestural experience to facilitate the learning of sign language vocabulary. With manual, binary coding for handshape, location, and movement, they found that cumulative accuracy was significantly higher for signs that were rated as highly iconic and resembled corresponding gestures (iconic signs with high overlaps) than for arbitrary signs, suggesting that learners use their gestural experience as gateways into the target sign language. The same research question can be answered using DTW: By comparing DTW distance scores across sign categories, one can examine whether the accuracy of learners’ signs – operationalized as the similarity between the learner’s sign and the target sign – is higher for iconic signs with high overlaps compared to iconic signs with low overlaps or arbitrary signs. In summary, DTW can be used to quantify similarity in various aspects of human language use, from co-speech gestures and silent gestures to sign languages.

### Future research

The majority of research on alignment in gesture form uses manual coding of gesture form similarity per gesture features such as handedness, handshape, movement, orientation, and position (e.g., Chui, 2014; Kimbara, 2006). Here, we showed that DTW can capture similarity for all features. However, it is not clear to what extent such “anatomical” gesture form similarity and DTW correlate with holistic “perceived” similarity. Also, we do not know whether each gesture feature is equally important for perceived similarity. For example, people may perceive a pair of gestures that are similar in handshape and movement as more similar than a gesture pair that is similar in orientation and handedness, even though the number of similar features is two for both pairs. This calls for a need to triangulate anatomical gesture form similarity, perceived gesture form similarity, and DTW to understand the characteristics of each measure so that researchers can choose the best measure or combination of measures for their theoretical interests (Punselie et al., 2024).

Another potential area for future research is developing a method to quantify the degree of similarity per gesture feature. Although we have observed a negative association between the DTW distance and manual coding for all features, our current approach does not specify *in which features* two gestures are similar or different. Establishing an automatic, kinematic approach that can specify the aligned and misaligned gesture features may be helpful if researchers are interested in understanding the degree of similarity in particular features.

## Conclusion

Growing interest in quantifying the degree of gesture form similarity using dynamic time warping (DTW) creates the need for a rigorous validation of DTW as a measure of gesture form similarity. To fill this gap, this study validated DTW with manual coding of gesture form similarity for two multimodal corpora of interaction and demonstrated its utility by showing a moderate negative correlation between the DTW distance and the manual gesture form similarity coding, suggesting that DTW offers a useful proxy for gesture form similarity. Furthermore, by inspecting pairs of gestures for which the DTW’s prediction and manual coding misaligned, we improved the predictive accuracy of DTW. Taken together, we have provided a first validation of DTW as a measure of gesture form similarity for spontaneous co-speech gestures with human-coded gesture form similarity ratings and a framework for extracting motion tracking data, preprocessing the time series data, and computing the DTW distance that quantifies the degree of similarity in gesture form. We hope that our DTW framework facilitates research on gesture and sign form similarity, and by being used complementarily with manual coding, it helps researchers reveal new insights into the mechanisms and functions of behavioral similarity.

## Figures and Tables

**Fig. 9:**
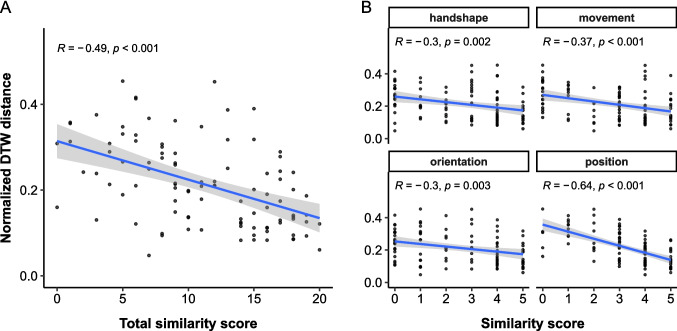
Normalized DTW distance by manual annotation of gesture form similarity. Each *dot* represents a pair of iconic gestures. **A** Relation between the normalized DTW distance and the cumulative gesture form similarity rating. The regression line from a linear model is shown as a *blue line* with its 95% confidence interval in the shared area. **B** Relation between the normalized DTW distance and gesture form similarity rating per feature

## Data Availability

Motion tracking data from MediaPipe are available on the OSF repository: https://osf.io/4bqys/overview?view_only=38e9579df0db41f9bfa5468577ad20ee. DTW distance scores, gesture form similarity ratings, and ELAN annotations containing speech transcripts, gesture annotations, and trial information are available on the GitHub repository: https://github.com/ShoAkamine/dtw_validation. Audio/video recordings for participants in Study 2 who agreed to share the identifiable data can be obtained upon request from this MPI for Psycholinguistics Archive repository: https://hdl.handle.net/1839/d9ff4aec-ae60-4435-8d18-18cc84688d0a.

## Appendix A Results for non-transformed similarity ratings

We computed a Pearson’s correlation coefficient between the normalized DTW distance scores from the modified pipeline and the cumulative manual similarity rating scores to examine whether the DTW distance is smaller for more similar gestures. To investigate the relation between the DTW distance and manual similarity ratings more in detail while controlling for differences in the mean DTW distance across dyads and items, we performed linear mixed-effects regression models on the DTW distance as a function of *z*-transformed cumulative gesture similarity rating scores with by-dyad and by-item varying intercepts. The cumulative gesture similarity rating scores were calculated by taking the sum of similarity ratings for handshape, movement, orientation, and position. We also examine, for each feature separately, whether the DTW distance is smaller for similar gesture pairs. Lastly, to determine which features have a stronger influence on the DTW distance, we included the similarity rating for each feature as separate fixed effects in a linear mixed-effects model to inspect the effect size of each feature *when controlling for the other features*.

A Pearson’s correlation coefficient revealed a moderate negative correlation between the DTW distance and the cumulative gesture form similarity rating ($r = -.49$, $p <.001$), indicating that the DTW distance is smaller for gesture pairs that are rated as more similar, as illustrated in Fig. 9A. A linear mixed-effects regression model with random intercepts for dyads and items also revealed a significant negative slope ($\beta = -0.049$, $SE =.009$, $t = -5.72$, $p <.001$), indicating that the negative association between the DTW distance and the cumulative gesture form similarity rating holds when accounting for differences in mean across dyads and items.

Another set of linear mixed-effects models, each including the *z*-transformed similarity rating for one of the four features as fixed effects, demonstrated significant negative slopes for all features: handshape ($\beta = -0.03$, $SE = 0.009$, $t = -3.21$, $p <.01$), movement ($\beta = -0.036$, $SE = 0.009$, $t = -3.94$, $p <.001$), orientation ($\beta = -0.031$, $SE = 0.009$, $t = -3.33$, $p <.01$), and position ($\beta = -0.064$, $SE = 0.008$, $t = -8.40$, $p <.001$). See Fig. 9B for the distribution of DTW distance scores.

We also performed a linear mixed-effects model with the *z*-transformed similarity rating for all four features as separate fixed effects to determine which features have a stronger influence on the DTW distance. The model showed that the normalized DTW distance is significantly lower for gesture pairs rated as highly similar in terms of position only ($\beta = -0.065$, $SE = 0.009$, $t = -6.85$, $p <.001$), but not in the other features (handshape: $\beta = -0.02$, $SE = 0.011$, $t = -1.85$, $p =.07$; movement: $\beta = 0.005$, $SE = 0.01$, $t = 0.46$, $p =.65$; orientation: $\beta = 0.009$, $SE = 0.012$, $t = 0.72$, $p =.47$), when controlling for the other three features. The fixed-effect correlation revealed a strong negative correlation between handshape and orientation (-0.61) and a moderate negative correlation between movement and position (-0.45), suggesting that when the negative slope for orientation or position is steeper, the slope for handshape or movement becomes flatter, respectively. The negative correlation in the fixed effects may stem from the fact that handshape and orientation are reflected in the relative fingertip positions, while movement and position are reflected in the wrist positions.

## Footnotes

1 Although we take manual coding of gesture form similarity as an approximation of gesture form similarity, it is likely not a perfectly reliable measure because it involves subjective judgments. However, it is among the widely used measures in the field, and we therefore use it as the ground truth.

2 The degree of improvement in $\beta$ from the original ($-0.027$) to the modified approach ($-0.032$) may appear small compared to the degree of improvement in correlation and thus may seem contradictory. However, these results are not contradictory and can be explained by the fact that correlation is influenced not only by the effect size ($\beta$) but also by the data’s variance ($SD^2$). For details, see “supplementary.md” file at https://github.com/ShoAkamine/dtw_validation.

## Code availability

R markdown files used for statistical analyses and Python notebooks used for MediaPipe motion tracking, time series data preprocessing, and DTW distance computations are available on the GitHub repository: https://github.com/ShoAkamine/dtw_validation.

## Declarations

### Open Practices Statement

MediaPipe motion tracking data for Studies 1 and 2 are available on the OSF repository: https://osf.io/4bqys/overview?view_only=38e9579df0db41f9bfa5468577ad20ee. Personal data (i.e., audio/video recordings) for Study 2 can be obtained upon request at https://hdl.handle.net/1839/d9ff4aec-ae60-4435-8d18-18cc84688d0a. R scripts for statistical analyses and Python scripts for preprocessing time series data and computing DTW distance are available on the GitHub repository: https://github.com/ShoAkamine/dtw_validation. None of the reported studies were preregistered.

## References

[CR1] Adistambha, K., Ritz, C. H., & Burnett, I. S. (2008). Motion classification using dynamic time warping. In *2008 IEEE 10th Workshop on Multimedia Signal Processing,* (pp. 622–627). 10.1109/MMSP.2008.4665151

[CR2] Akamine, S., Dingemanse, M., Meyer, A., & Özyürek, A. (2025). *Video-mediated multimodal referential communication dataset*. MPI for Psycholinguistics Archive, 2025. Retrieved from https://hdl.handle.net/1839/d9ff4aec-ae60-4435-8d18-18cc84688d0a

[CR3] Akamine, S., Ghaleb, E., Rasenberg, M., Fernandez, R., Meyer, A., & Özyürek, A. (2024). Speakers align both their gestures and words not only to establish but also to maintain reference to create shared labels for novel objects in interaction. In *Proceedings of the 46th Annual Meeting of the Cognitive Science Society,* Rotterdam, Netherlands.

[CR4] Bates, D., Mächler, M., Bolker, B. M., & Walker, S. C. (2015). Fitting linear mixed-effects models using lme4. *Journal of Statistical Software,**67*(1), 1–48. arXiv: 1406.5823, 10.18637/jss.v067.i01

[CR5] Bautista, M. Á., Hernández-Vela, A., Ponce, V., Perez-Sala, X., Baró, X., Pujol, O., . . . Escalera, S. (2013). Probability-based dynamic time warping for gesture recognition on RGB-D data. In X. Jiang, O. R. P. Bellon, D. Goldgof, & T. Oishi (Eds.), *Advances in Depth Image Analysis and Applications* (Vol. 7854, pp. 126–135). 10.1007/978-3-642-40303-3_14

[CR6] Beecks, C., Hassani, M., Hinnell, J., Schüller, D., Brenger, B., Mittelberg, I., & Seidl, T. (2015). Spatiotemporal similarity search in 3D motion capture gesture streams. In C. Claramunt, M. Schneider, R. C.-W. Wong, L. Xiong, W.-K. Loh, C. Shahabi, & K.-J. Li (Eds.), *Advances in Spatial and Temporal Databases* (pp. 355–372). 10.1007/978-3-319-22363-6_19

[CR7] Bergmann, K., & Kopp, S. (2012). Gestural alignment in natural dialogue. In *Proceedings of the Annual Meeting of the Cognitive Science Society,**34*(34). Retrieved November 13, 2023, from https://escholarship.org/uc/item/73z0q063

[CR8] Branigan, H. P., Pickering, M. J., & Cleland, A. A. (2000). Syntactic co-ordination in dialogue. *Cognition,**75*(2), B13–B25. 10.1016/S0010-0277(99)00081-5 10771277 10.1016/s0010-0277(99)00081-5

[CR9] Brehm, L., & Alday, P. M. (2022). Contrast coding choices in a decade of mixed models. *Journal of Memory and Language,**125*, Article 104334. 10.1016/j.jml.2022.104334

[CR10] Brennan, S. E., & Clark, H. H. (1996). Conceptual pacts and lexical choice in conversation. *Journal of Experimental Psychology. Learning, Memory, and Cognition,**22*(6), 1482–1493. 10.1037/0278-7393.22.6.1482 8921603 10.1037//0278-7393.22.6.1482

[CR11] Celebi, S., Aydin, A. S., Temiz, T. T., & Arici, T. (2013). Gesture recognition using skeleton data with weighted dynamic time warping. In *Proceedings of the International Conference on Computer Vision Theory and Applications,* (pp. 620–625). 10.5220/0004217606200625

[CR12] Chartrand, T. L., & Bargh, J. A. (1999). The chameleon effect: The perception–behavior link and social interaction. *Journal of Personality and Social Psychology,**76*(6), 893–910. 10.1037/0022-3514.76.6.893 10402679 10.1037//0022-3514.76.6.893

[CR13] Chui, K. (2014). Mimicked gestures and the joint construction of meaning in conversation. *Journal of Pragmatics,**70*, 68–85. 10.1016/j.pragma.2014.06.005

[CR14] Dau, H. A., Bagnall, A., Kamgar, K., Yeh, C.-C. M., Zhu, Y., Gharghabi, S., . . . Keogh, E. (2019). The UCR time series archive. arXiv: 1810.07758, [cs] 10.48550/arXiv.1810.07758

[CR15] Dau, H. A., Keogh, E., Kamgar, K., Yeh, C.-C. M., Zhu, Y., Gharghabi, S., . . . Hexagon-ML. (2018). The UCR time series classification archive. Retrieved from https://www.cs.ucr.edu/~eamonn/time_series_data_2018/

[CR16] Ghaleb, E., Burenko, I., Rasenberg, M., Pouw, W., Uhrig, P., Holler, J., . . . Fernández, R. (2024). Co-speech gesture detection through multi-phase sequence labeling. (pp. 4007–4015). In *Proceedings of the IEEE/CVF Winter Conference on Applications of Computer Vision*. Retrieved May 19, 2025, from https://openaccess.thecvf.com/content/WACV2024/html/Ghaleb_CoSpeech_Gesture_Detection_Through_MultiPhase_Sequence_Labeling_WACV_2024_paper.html

[CR17] Giorgino, T. (2009). Computing and visualizing dynamic time warping alignments in R: The dtw package. *Journal of Statistical Software,**31*, 1–24. 10.18637/jss.v031.i07

[CR18] Holler, J., & Wilkin, K. (2011). Co-speech gesture mimicry in the process of collaborative referring during face-to-face dialogue. *Journal of Nonverbal Behavior,**35*(2), 133–153. 10.1007/s10919-011-0105-6

[CR19] Karadöller, D. Z., Sümer, B., & Özyürek, A. (2024). First-language acquisition in a multimodal language framework: Insights from speech, gesture, and sign. *First Language,**01427237241290678*. 10.1177/01427237241290678

[CR20] Kimbara, I. (2006). On gestural mimicry. *Gesture,**6*(1), 39–61. 10.1075/gest.6.1.03kim

[CR21] Ko, M. H., West, G., Venkatesh, S., & Kumar, M. (2005). Online context recognition in multisensor systems using dynamic time warping. In *2005 International Conference on Intelligent Sensors, Sensor Networks and Information Processing,* (pp. 283–288). 10.1109/ISSNIP.2005.1595593

[CR22] Kopp, S., & Bergmann, K. (2013). Automatic and strategic alignment of co-verbal gestures in dialogue. In I. Wachsmuth, J. de Ruiter, P. Jaecks, & S. Kopp (Eds.), *Alignment in communication: Towards a new theory of communication* (pp. 87–108). 10.1075/ais.6.05kop

[CR23] Lakin, J. L., & Chartrand, T. L. (2003). Using nonconscious behavioral mimicry to create affiliation and rapport. *Psychological Science,**14*(4), 334–339. 10.1111/1467-9280.14481 12807406 10.1111/1467-9280.14481

[CR24] Levelt, W. J. M., & Kelter, S. (1982). Surface form and memory in question answering. *Cognitive Psychology,**14*(1), 78–106. 10.1016/0010-0285(82)90005-6

[CR25] Liu, J., Wang, Z., Zhong, L., Wickramasuriya, J., & Vasudevan, V. (2009). uWave: Accelerometer-based personalized gesture recognition and its applications. In *2009 IEEE International Conference on Pervasive Computing and Communications,* (pp. 1–9). 10.1109/PERCOM.2009.4912759

[CR26] Lugaresi, C., Tang, J., Nash, H., McClanahan, C., Uboweja, E., Hays, M., ..., Grundmann, M. (2019). MediaPipe: A framework for building perception pipelines. arXiv: 1906.08172 [cs] 10.48550/arXiv.1906.08172

[CR27] Mezari, A., & Maglogiannis, I. (2017). Gesture recognition using symbolic aggregate approximation and dynamic time warping on motion data. In *Proceedings of the 11th EAI International Conference on Pervasive Computing Technologies for Healthcare,* (pp. 342–347). 10.1145/3154862.3154 927

[CR28] Müller, M. (2007). Dynamic time warping. In *Information retrieval for music and motion,* (pp. 69–84). 10.1007/978-3-540-74048-3_4

[CR29] Oben, B., & Brône, G. (2016). Explaining interactive alignment: A multimodal and multifactorial account. *Journal of Pragmatics,**104*, 32–51. 10.1016/j.pragma.2016.07.002

[CR30] Pearson, L., Nuttall, T., & Pouw, W. (2024). The co-structuring of gesture-vocal dynamics: An exploration in Karnatak music performance. 10.31234/osf.io/npm96. 10.1111/cogs.70137 PMC12665335 41319266

[CR31] Pouw, W., & Akamine, S. (2025). Using Media-Pipe for full-body tracking, masking, blurring, and movement tracing. Retrieved May 6, 2025, from https://github.com/ WimPouw/envisionBOX_modulesWP/tree/main/Mediapipe_Optional_Masking

[CR32] Pouw, W., de Wit, J., Bögels, S., Rasenberg, M., Milivojevic, B., & Özyürek, A. (2021). Semantically related gestures move alike: Towards a distributional semantics of gesture kinematics. In V. G. Duffy (Ed.), *Digital Human Modeling and Applications in Health, Safety, Ergonomics and Risk Management. Human Body, Motion and Behavior* (pp. 269–287). 10.1007/978-3-030-77817-0_20

[CR33] Pouw, W., Dingemanse, M., Motamedi, Y., & Özyürek, A. (2021). A systematic investigation of gesture kinematics in evolving manual languages in the lab. *Cognitive Science,**45*(7), e13014. 10.1111/cogs.13014 34288069 10.1111/cogs.13014 PMC8365719

[CR34] Pouw, W., & Dixon, J. A. (2019). Gesture networks: Introducing dynamic time warping and network analysis for the kinematic study of gesture ensembles. *Discourse Processes,**57*(4), 301–319. 10.1080/0163853X.2019.1678967

[CR35] Pouw, W., Yung, B., Shaikh, S. A., Trujillo, J., Rueda-Toicen, A., Melo, G. de, & Owoyele, B. (2025). EnvisionHGdetector: A computational framework for co-speech gesture detection, kinematic analysis, and interactive visualization. 10.31234/osf.io/psg5f_v1

[CR36] Punselie, S., McLean, B., & Dingemanse, M. (2024). The anatomy of iconicity: Cumulative structural analogies underlie objective and subjective measures of iconicity. *Open Mind,**8*, 1191–1212. 10.1162/opmi_a_00162 39439590 10.1162/opmi_a_00162 PMC11495960

[CR37] R Core Team. (2024). R: A language and environment for statistical computing. Vienna, Austria. Retrieved from https://www.R-project.org

[CR38] Raihan, T. (2017). Predicting US recessions: A dynamic time warping exercise in economics. *Social Science Research Network,**3047649*. 10.2139/ssrn.3047649

[CR39] Rakthanmanon, T., Campana, B., Mueen, A., Batista, G., Westover, B., Zhu, Q., . . . Keogh, E. (2013). Addressing big data time series: Mining trillions of time series subsequences under dynamic time warping. *ACM Transactions on Knowledge Discovery from Data,**7*(3), 10:1–10:31. 10.1145/2500489 PMC6790126 31607834

[CR40] Rasenberg, M. (2023). Mutual understanding from a multimodal and interactional perspective (Doctoral dissertation, Radboud University, Nijmegen, Netherlands).

[CR41] Rasenberg, M., Özyürek, A., Bögels, S., & Dingemanse, M. (2022). The primacy of multimodal alignment in converging on shared symbols for novel referents. *Discourse Processes,**59*(3), 209–236. 10.1080/0163853X.2021.1992235

[CR42] Ratanamahatana, C., & Keogh, E. [E.]. (2004). Everything you know about dynamic time warping is wrong. In *3rd International Workshop on Mining Temporal and Sequential Data (TDM-04)*.

[CR43] Ratanamahatana, C., & Keogh, E. [Eamonn]. (2005). Three myths about dynamic time warping data mining. In *Proceedings of the 2005 SIAM International Conference on Data Mining,* (pp. 506–510). 10.1137/1.9781611972757.50

[CR44] Reyes, M., Domínguez, G., & Escalera, S. (2011). Feature weighting in dynamic time warping for gesture recognition in depth data. In *2011 IEEE International Conference on Computer Vision Workshops (ICCV Workshops),* (pp. 1182–1188). 10.1109/ICCVW.2011.613038

[CR45] Silva, D. F., Batista, G. E. A. P. A., & Keogh, E. (2016). Prefix and suffix invariant dynamic time warping. In *2016 IEEE 16th International Conference on Data Mining (ICDM),* (pp. 1209–1214). 10.1109/ICDM.2016.0161

[CR46] Tormene, P., Giorgino, T., Quaglini, S., & Stefanelli, M. (2009). Matching incomplete time series with dynamic time warping: An algorithm and an application to post-stroke rehabilitation. *Artificial Intelligence in Medicine,**45*(1), 11–34. 10.1016/j.artmed.2008.11.007 10.1016/j.artmed.2008.11.007 19111449

[CR47] Trujillo, J. P., Dideriksen, C., Tylén, K., Christiansen, M. H., & Fusaroli, R. (2023). The dynamic interplay of kinetic and linguistic coordination in Danish and Norwegian conversation. *Cognitive Science,**47*(6), Article e13298. 10.1111/cogs.13298 10.1111/cogs.13298 37303224

[CR48] Wittenburg, P., Brugman, H., Russel, A., Klassmann, A., Sloetjes, H. (2006). ELAN: A professional framework for multimodality research. In *Proceedings of the Fifth International Conference on Language Resources and Evaluation (LREC’06)*. LREC 2006, Genoa, Italy: European Language Resources Association (ELRA). Retrieved August 24, 2023, from http://www.lrec-conf.org/proceedings/lrec2006/pdf/153_pdf.pdf

[CR49] Wu, R., Der, A., & Keogh, E. J. (2021). When is early classification of time series meaningful? *IEEE Transactions on Knowledge and Data Engineering,* pp. 1–1. arXiv: 2102.11487, 10.1109/TKDE.2021.3108580

